# Supplementary material for: Primary decidual zone formation requires Scribble for pregnancy success in mice
Source: Nat Commun. 2019 Nov 28;10:5425. doi: 10.1038/s41467-019-13489-4 (PMC6882879; doi:10.1038/s41467-019-13489-4)
Supplement: Supplementary file 1 — Supplementary Information [file 41467_2019_13489_MOESM1_ESM.pdf]

Supplementary Information:

Primary Decidual Zone formation requires Scribble for pregnancy success in mice

Yuan et al.

Supplementary Table 1. Summary of pregnancy outcome.

genotype	No. of females with plugs	No. of females with pregnancy failures (%)	Average litter sizes (Mean ± SD)
§*Scrib*$^{f/f}$	16	1/16 (6)	8.1 ± 1.4
#*Scrib*$^{f/f}$*Ltf*$^{Cre/+}$	39	11/39 (28)	7.8 ± 2.9
¶*Scrib*$^{f/f}$*Pgr*$^{Cre/+}$	35	20/35 (57)	4.7 ± 1.9***

§Twelve *Scrib*$^{f/f}$ females were mated and 3 of them were mated 2-3 times.

#Twenty *Scribf*^{f}*Ltf*$^{Cre/+}$ females were mated with 8 of them 2-3 times.

¶Twenty-four *Scrib*$^{f/f}$*Pgr*$^{Cre/+}$ females were mated with 9 of them 2-4 times.

Average litter sizes are shown as mean ± SD. Litter sizes are calculated only from females who gave live birth. The data were analyzed by One-way ANOVA followed by Bonferroni post-hoc test. ***P < 0.001.

Supplementary Table 2. List of primary and secondary antibodies for IF and WB.

Primary Antibodies	Dilution	Company	Cat#
Rabbit polyclonal anti-Scribble	1:500 for IF	Santa Cruz	sc-28737
Rabbit anti-Scribble	1:1000 for WB	Custom made by Mireille Montcouquiol	N/A
Rat monoclonal anti-Vangl2	1:1000 for IF<br>1:10000 for WB	Custom made by JP Borg	N/A
Rabbit polyclonal anti-ERα (Esr1)	1:300 for IF	Santa Cruz	sc-542
Rabbit monoclonal anti-Progesterone Receptor A/B	1:300 for IF	Cell Signaling Technology	8757S
Rabbit monoclonal anti-E-Cadherin	1:300 for IF<br>1:100 for 3D	Cell Signaling Technology	3195S
Goat polyclocal anti-β-catenin	1:300 for IF	Santa Cruz	sc-1496
Rabbit monoclonal anti-Ki67	1:300 for IF	Thermo Fisher	RM-9106-S
Rabbit polyclonal anti-pHH3	1:300 for IF	Cell Signaling Technology	9701S
Rabbit anti-FLK1	1:300 for IF	Neomarker	RB-9239
Rat monoclonal anti-Krt8(CK8)	1:1000 for IF	DSHB	TROMA-I
Rat monoclonal anti-ZO-1	1:300 for IF	Thermo Fisher	14-9776-82
Rabbit monoclonal anti-Sav1	1:300 for IF	Cell Signaling Technology	13301
Rabbit monoclonal anti-Lats1	1:300 for IF	Cell Signaling Technology	3477
Rat monoclonal anti-CD45	1:300 for IF	Biolegend/Labome	13301
Rabbit monoclonal anti-Cleaved Caspase-3	1:300 for IF	Cell Signaling Technology	9661S
Rabbit polyclonal anti-YAP	1:300 for IF	Cell Signaling Technology	4912S
Rabbit polyclonal anti-MST1	1:1000 for WB	Cell Signaling Technology	3682S
Rabbit polyclonal anti-MST2	1:1000 for WB	Cell Signaling Technology	3952S
Rabbit monoclonal anti-phospho-MST1/MST2	1:1000 for WB	Cell Signaling Technology	49332S
Goat polyclonal anti-β-Actin	1:1000 for WB	Santa Cruz	sc-1615
**Secondary Antibodies**	**Dilution**	**Company**	**Cat#**
Alexa Fluor® 488 AffiniPure Donkey Anti-Rabbit IgG (H+L)	1:300 for IF	Jackson ImmunoResearch	711-545-152
Alexa Fluor® 594 AffiniPure Donkey Anti-Rabbit IgG (H+L)	1:300 for IF and 3D	Jackson ImmunoResearch	711-585-152
Alexa Fluor® 594 AffiniPure Donkey Anti-Rat IgG (H+L)	1:300 for IF	Jackson ImmunoResearch	712-585-150
Cy2 AffiniPure Donkey Anti-Rat IgG (H+L)	1:300 for IF	Jackson ImmunoResearch	712-225-153
Cy3 AffiniPure Donkey Anti-Goat IgG (H+L)	1:300 for IF	Jackson ImmunoResearch	705-165-147
Peroxidase AffiniPure Donkey Anti-Rabbit IgG (H+L)	1:5000 for WB	Jackson ImmunoResearch	711-035-152
Peroxidase AffiniPure Donkey Anti-Rat IgG (H+L)	1:5000 for WB	Jackson ImmunoResearch	712-035-150
Peroxidase AffiniPure Donkey Anti-Goat IgG (H+L)	1:5000 for WB	Jackson ImmunoResearch	705-035-147

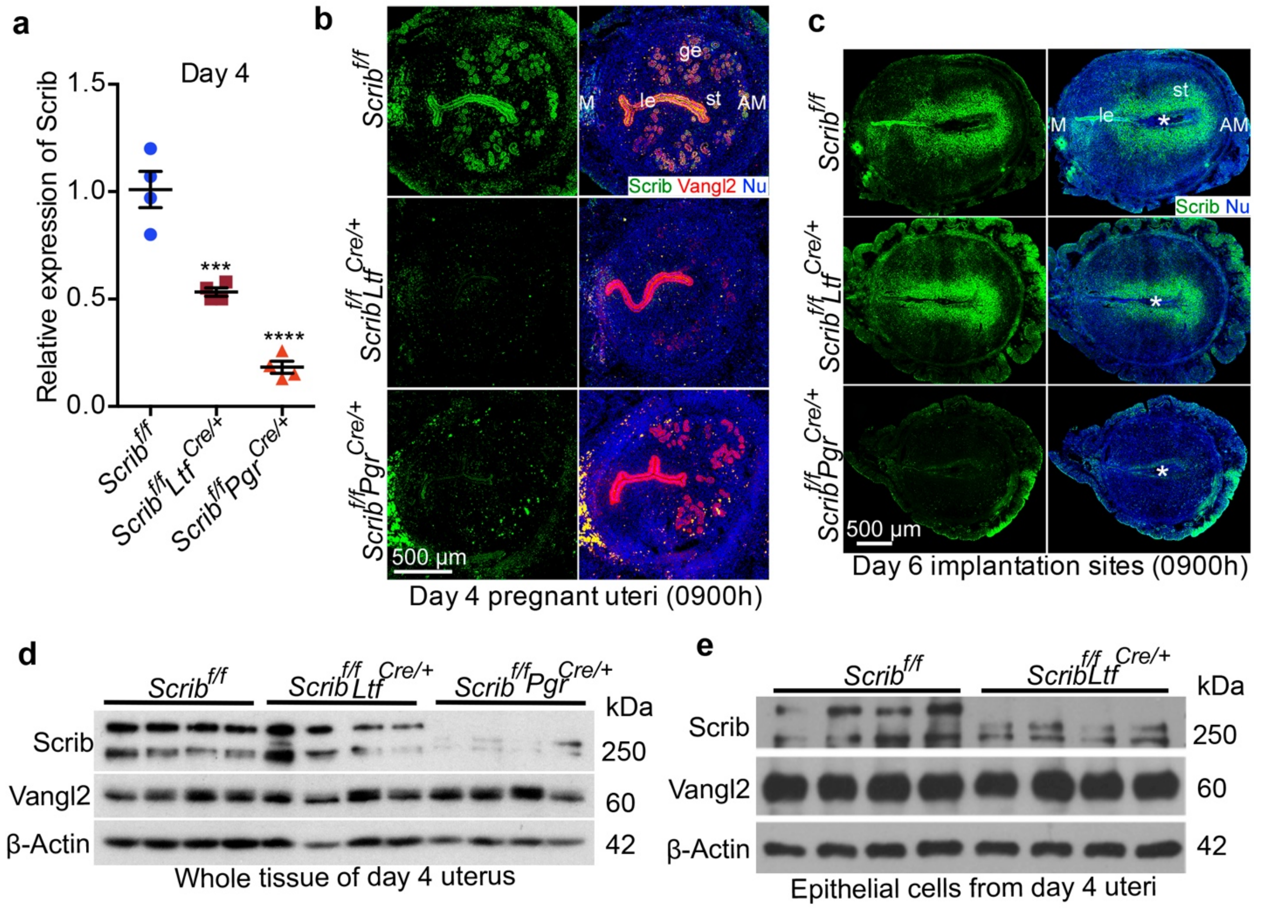

Supplementary Figure 1 | Scrib is efficiently deleted in respective uterine cell types of *$Scrib^{f/f}Ltf^{cre/+}$* and *$Scrib^{f/f}Pgr^{cre/+}$* females. a Q-PCR of Scrib in day4 uteri of *$Scrib^{f/f}$*, *$Scrib^{f/f}Ltf^{cre/+}$* and *$Scrib^{f/f}Pgr^{cre/+}$* females. n = 4 biologically independent samples for each genotype. Data are shown as mean ± SEM. ***$P < 0.001$, **** $P < 0.0001$ by One-way ANOVA followed by Bonferroni post-hoc test. **b** IF of Scrib and Vangl2 in day4 uteri of each genotype. Scale bar: 500 μm. M: mesometrial pole, AM: anti-mesometrial pole, le: luminal epithelium, st: stroma. **c** IF of Scrib in day 6 uteri of each genotype. Scale bar: 500 μm. Asterisks indicate the location of embryos. M: mesometrial pole, AM: anti-mesometrial pole, le: luminal epithelium, st: stroma. **d** Western blotting of Scrib and Vangl2 in day 4 uteri of each genotype. β-Actin was used as internal control. See also Supplementary Figure 10. **e** Western blotting of Scrib and Vangl2 in day4 epithelium of each genotype. β-Actin was used as internal control. See also Supplementary Figure 11.

Each image is a representative from at least 3 independent experiments.

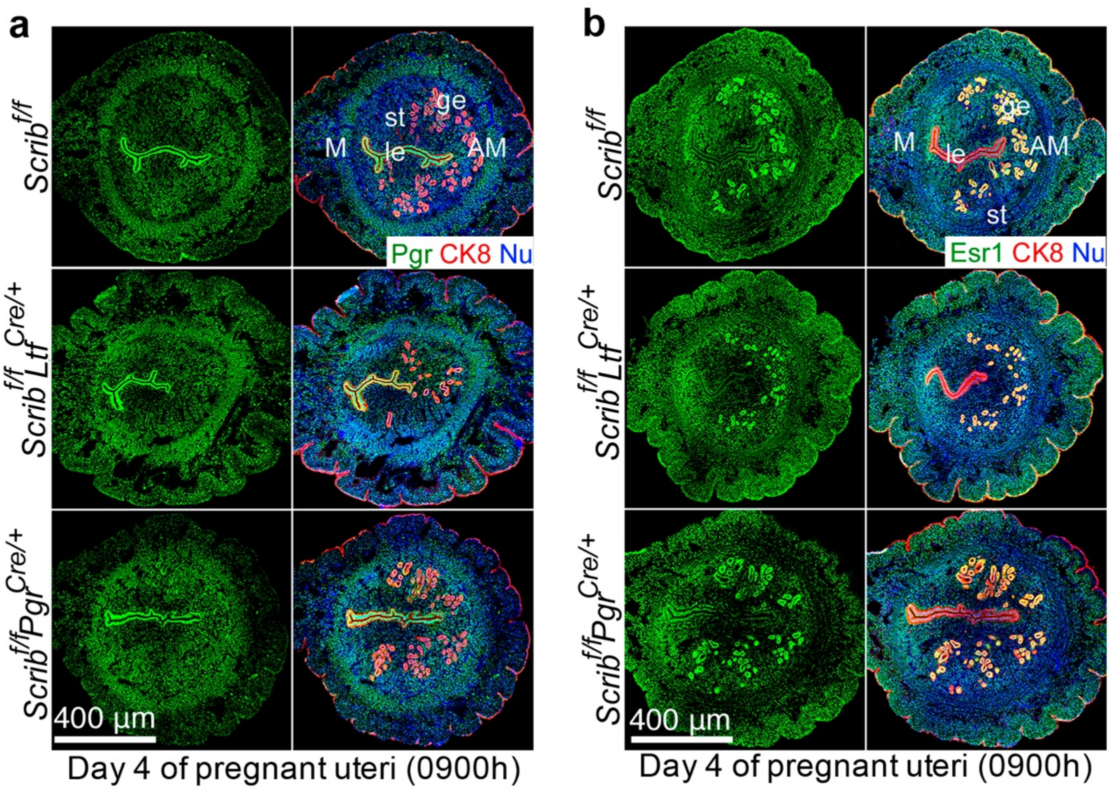

Supplementary Figure 2 | Uterine receptivity marker gene expression is comparable in *Scrib$^{f/f}$*, *Scrib$^{f/f}$Ltf$^{cre/+}$* and *Scrib$^{f/f}$Pgr$^{cre/+}$* females. a, b IF of Pgr and Esr1 in day4 uteri of *Scrib$^{f/f}$*, *Scrib$^{f/f}$Ltf$^{cre/+}$* and *Scrib$^{f/f}$Pgr$^{cre/+}$* mice. Scale bar: 400 µm.

M: mesometrial pole, AM: anti-mesometrial pole, le: epithelium, st: stroma, ge: glandular epithelium.

Each image is a representative from at least 3 independent experiments.

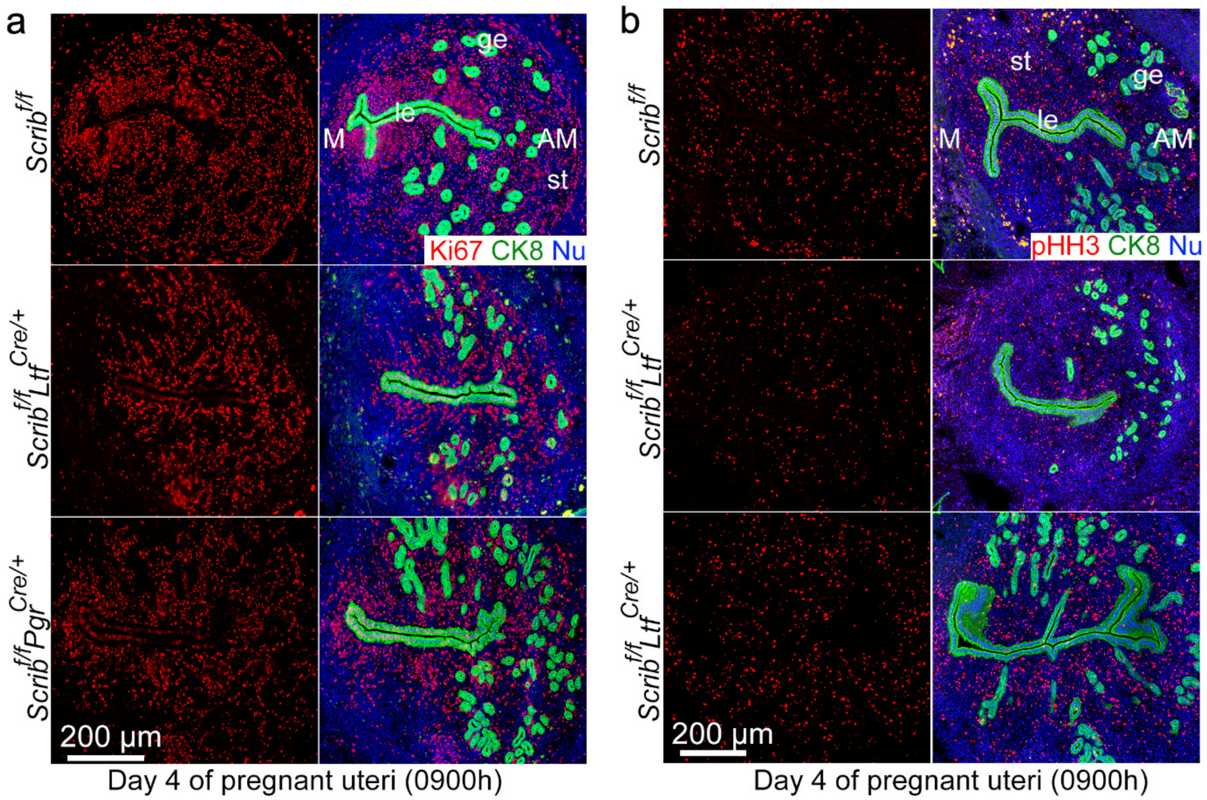

Supplementary Figure 3 I Stromal cell proliferation is comparable between *Scrib$^{f/f}$*, *Scrib$^{f/f}$Ltf$^{cre/+}$* and *Scrib$^{f/f}$Pgr$^{cre/+}$* females in Day 4 uteri. a,b IF of Ki67 and pHH3 in day 4 uteri of *Scrib$^{f/f}$, Scrib$^{f/f}$Ltf$^{cre/+}$* and *Scrib$^{f/f}$Pgr$^{cre/+}$* mice. Scale bar: 200 µm.

M: mesometrial pole, AM: anti-mesometrial pole, le: epithelium, st: stroma, ge: glandular epithelium.

Each image is a representative from at least 3 independent experiments.

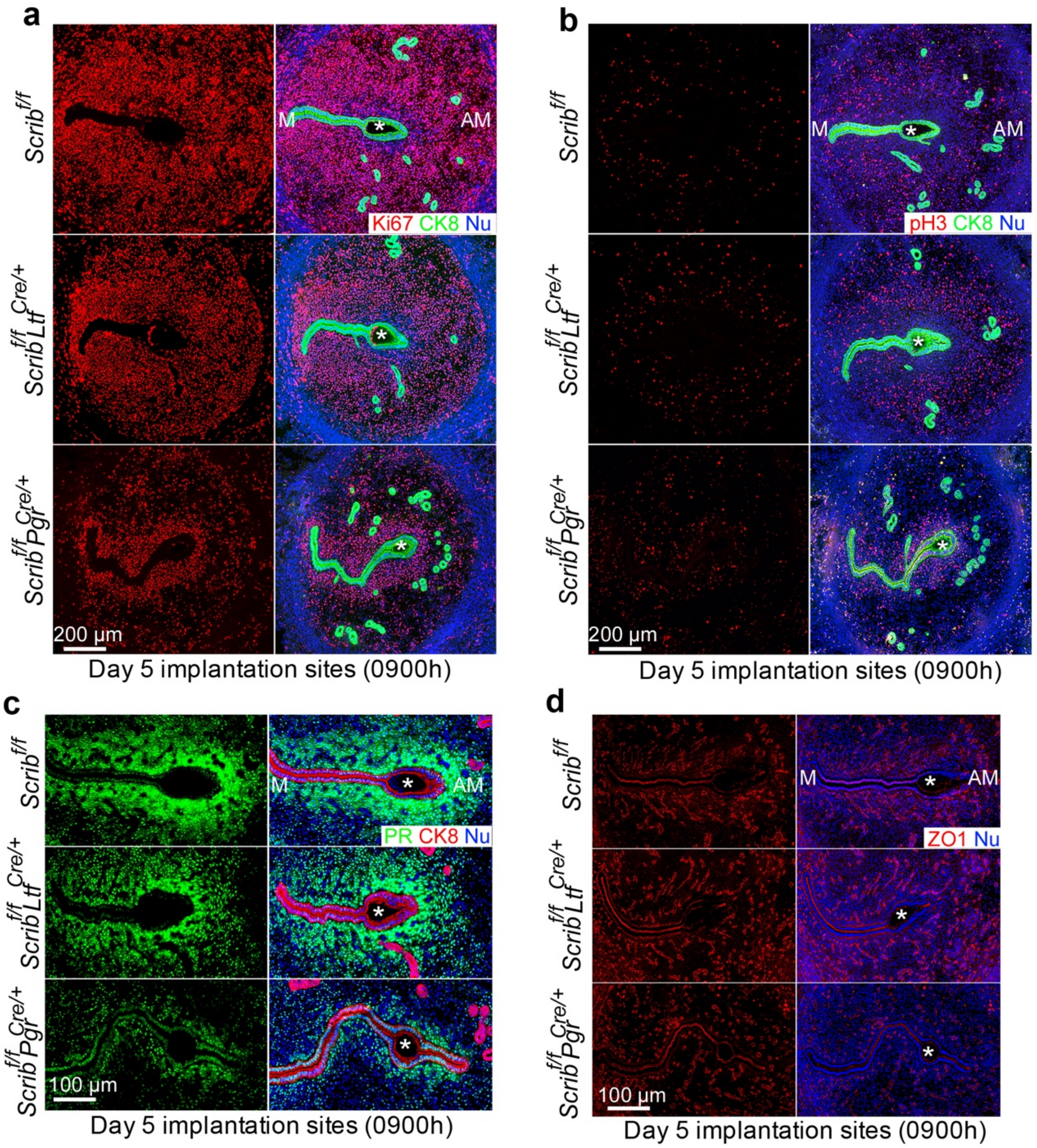

Supplementary Figure 4 | Stromal cell proliferation is aberrant in *Scrib*$^{f/f}$*Pgr*$^{cre/+}$ females after implantation. a, b IF of Ki67 and pHH3 on day 5 morning implantation sites of *Scrib*$^{f/f}$, *Scrib*$^{f/f}$*Ltf*$^{cre/+}$ and *Scrib*$^{f/f}$*Pgr*$^{cre/+}$ mice. Scale bar: 200 µm. **c** IF colocalization of Pgr and CK8 in *Scrib*$^{f/f}$, *Scrib*$^{f/f}$*Ltf*$^{cre/+}$ and *Scrib*$^{f/f}$*Pgr*$^{cre/+}$ mice on day 5 morning implantation site. **d** IF colocalization of ZO-1 in *Scrib*$^{f/f}$, *Scrib*$^{f/f}$*Ltf*$^{cre/+}$ and *Scrib*$^{f/f}$*Pgr*$^{cre/+}$ mice on day 5 morning implantation sites. Scale bar: 100 µm.

Asterisks indicate the location of blastocysts. M: mesometrial pole, AM: anti-mesometrial pole, le: luminal epithelium, st: stroma, ge: glandular epithelium. Each image is a representative from at least 3 independent experiments.

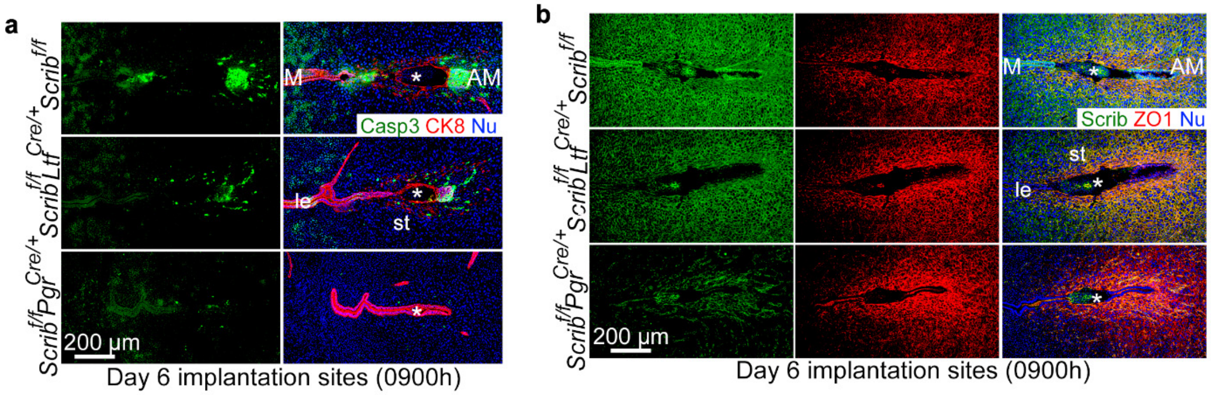

Supplementary Figure 5 l Establishment of PDZ is derailed in $Scrib^{f/f}Pgr^{cre/+}$ mice. a IF of Caspase3 and CK8 in day 6 implantation sites of $Scrib^{f/f}$, $Scrib^{f/f}Ltf^{cre/+}$ and $Scrib^{f/f}Pgr^{cre/+}$ mice. Scale bar: 200 μm. **b** IF colocalization of Scrib and ZO-1 in day 6 implantation sites *of* $Scrib^{f/f}$, $Scrib^{f/f}Ltf^{cre/+}$ and $Scrib^{f/f}Pgr^{cre/+}$ mice. Scale bar: 200 μm.

Asterisks indicate the location of embryos. M: mesometrial pole, AM: anti-mesometrial pole, le: luminal epithelium, st: stromaEach image is a representative from at least 3 independent experiments.

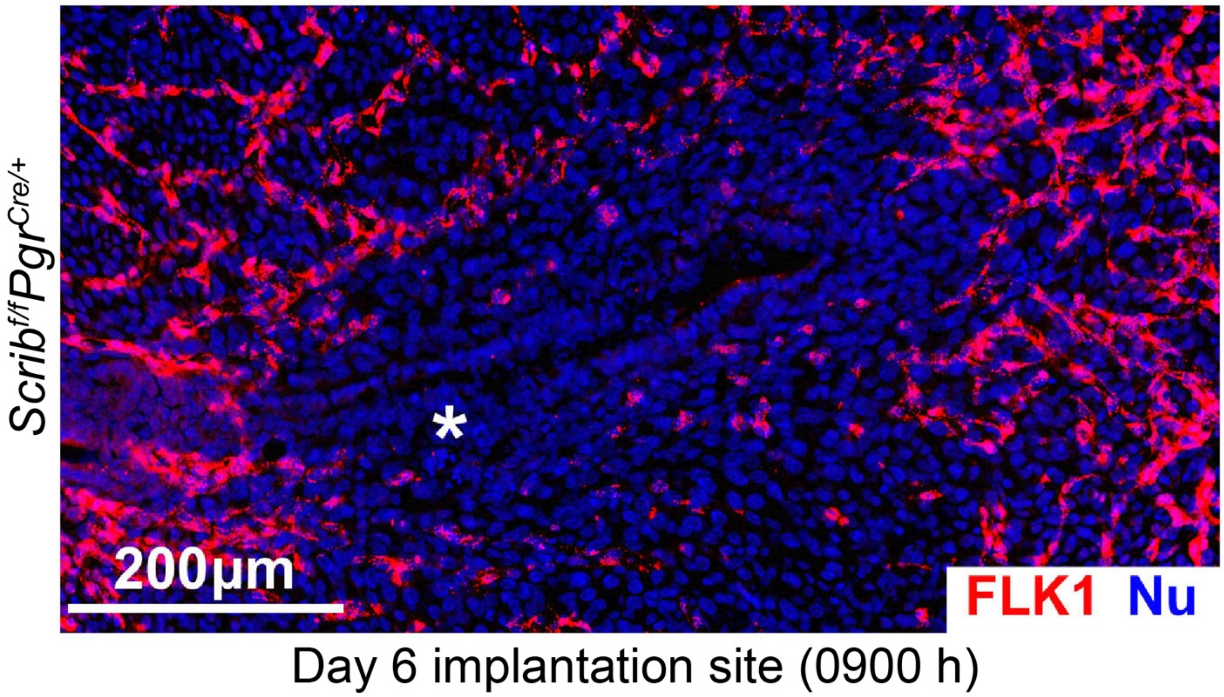

Supplementary Figure 6 | IF of FLK1 in a day 6 implantation site of *Scrib*$^{f/f}$*Pgr*$^{cre/+}$ mice shows blood vessels invaded the primary decidual zone. Scale bar: 200 µm. Asterisk indicates the location of the embryo.

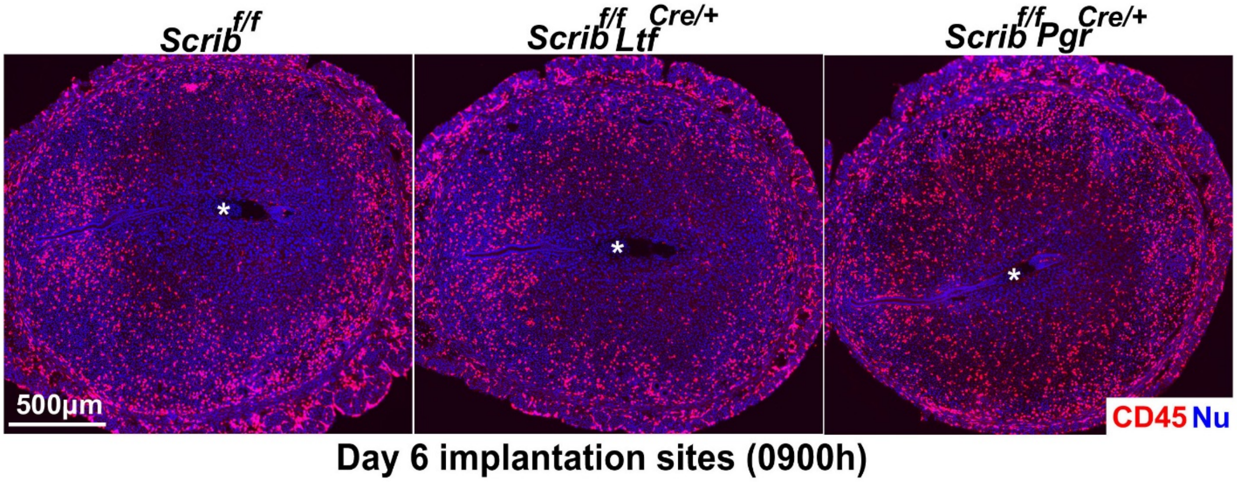

Supplementary Figure 7 | Immunostaining of CD45 in day 6 implantation sites of *Scrib*$^{f/f}$, *Scrib*$^{f/f}$*Ltf*$^{cre/+}$ and *Scrib*$^{f/f}$*Pgr*$^{cre/+}$ mice. Scale bar: 500 μm. Asterisks indicate the location of embryos. Nu, Nuclei.

Each image is a representative from at least 3 independent experiments.

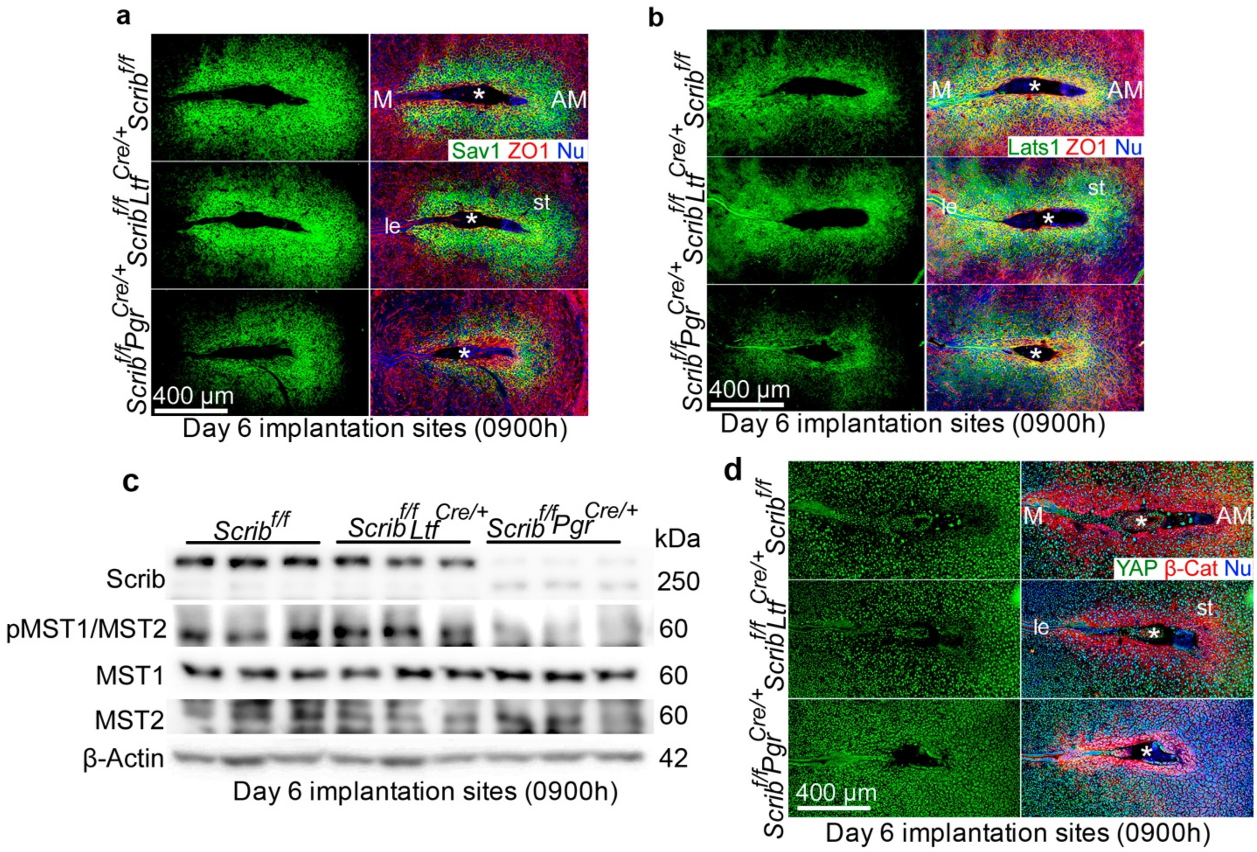

Supplementary Figure 8 | Establishment of PDZ is derailed in *Scrib^f/f^Pgr^cre/+^* mice via compromised Hippo signaling. **a** IF colocalization of Sav1 and ZO-1 in day 6 implantation sites of *Scrib$^{f/f}$, Scrib$^{f/f}$Ltf$^{cre/+}$* and *Scrib$^{f/f}$Pgr$^{cre/+}$* mice. Scale bar: 400 μm. **b** IF colocalization of Lats1 and ZO-1 in day 6 implantation sites of *Scrib$^{f/f}$, Scrib$^{f/f}$Ltf$^{cre/+}$* and *Scrib$^{f/f}$Pgr$^{cre/+}$* mice. Scale bar: 400 μm. **c** Western blotting of Scrib, pMST1/MST2, MST1 and MST2 in endometrium of day 6 implantation sites (Removed muscular layers) of each genotype. β-Actin was used as internal control. The same samples were run in multiple gels/blots in parallel. See also Supplementary Figure 12. **d** IF of YAP and β-catenin (a marker of PDZ) in day 6 implantation sites from females with each indicated genotype. Scale bar: 400 μm.

Asterisks indicate the location of embryos. M: mesometrial pole, AM: anti-mesometrial pole, le: luminal epithelium, st: stroma.

Each image is a representative from at least 3 independent experiments.

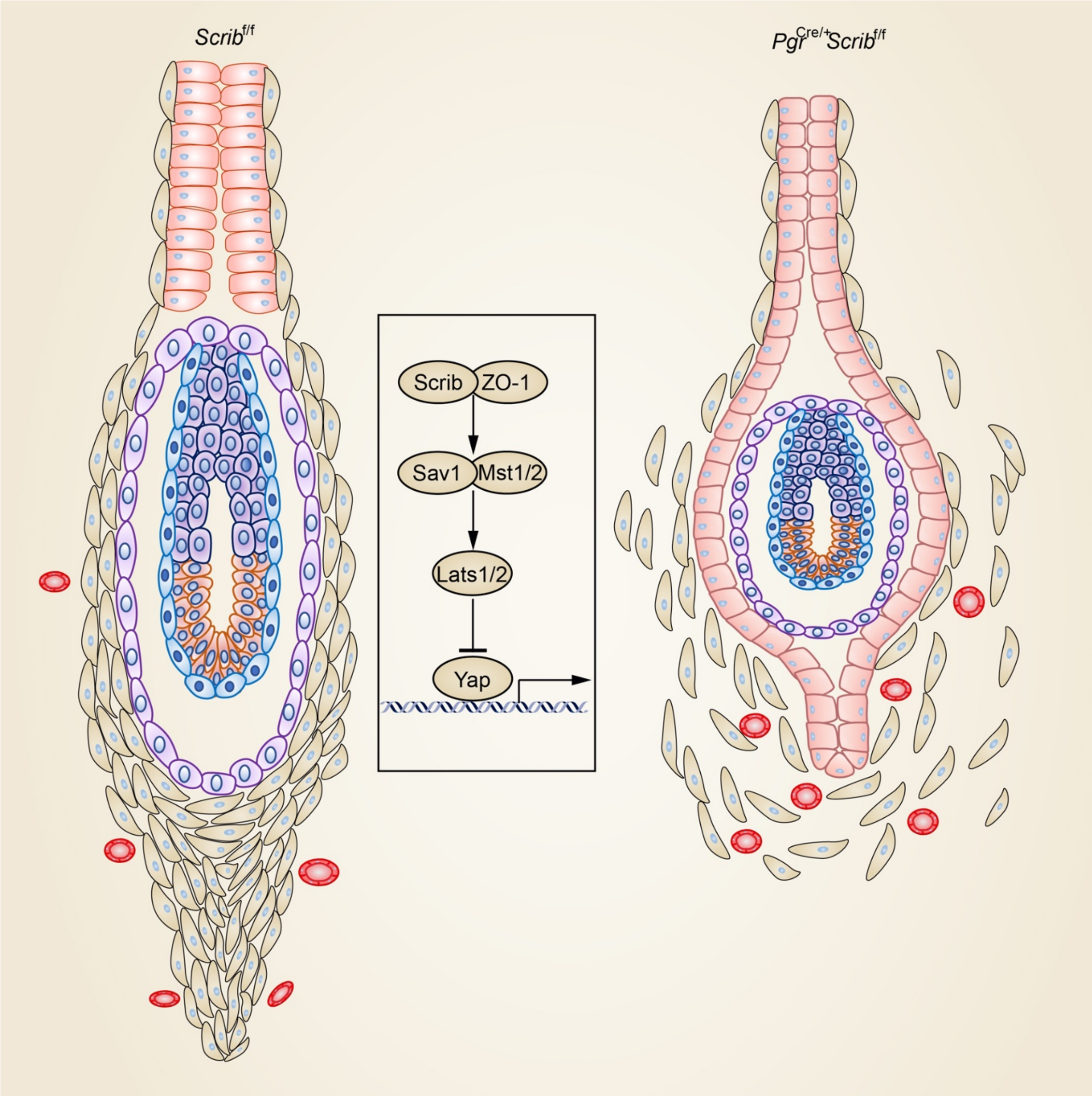

Supplementary Figure 9 l A proposed scheme of Scribble's role in PDZ formation. As a scaffold protein, Scrib not only participates in PCP activity in epithelial cells by interacting with Vanlg2, but also directs PDZ formation in the stroma through the Hippo signaling pathway. With Scrib ablation in the stroma, PDZ formation is characterized by delayed ZO-1 expression with inactivation of Hippo signaling. Defective PDZ formation leads to aberrant implantation chamber (crypt) formation.

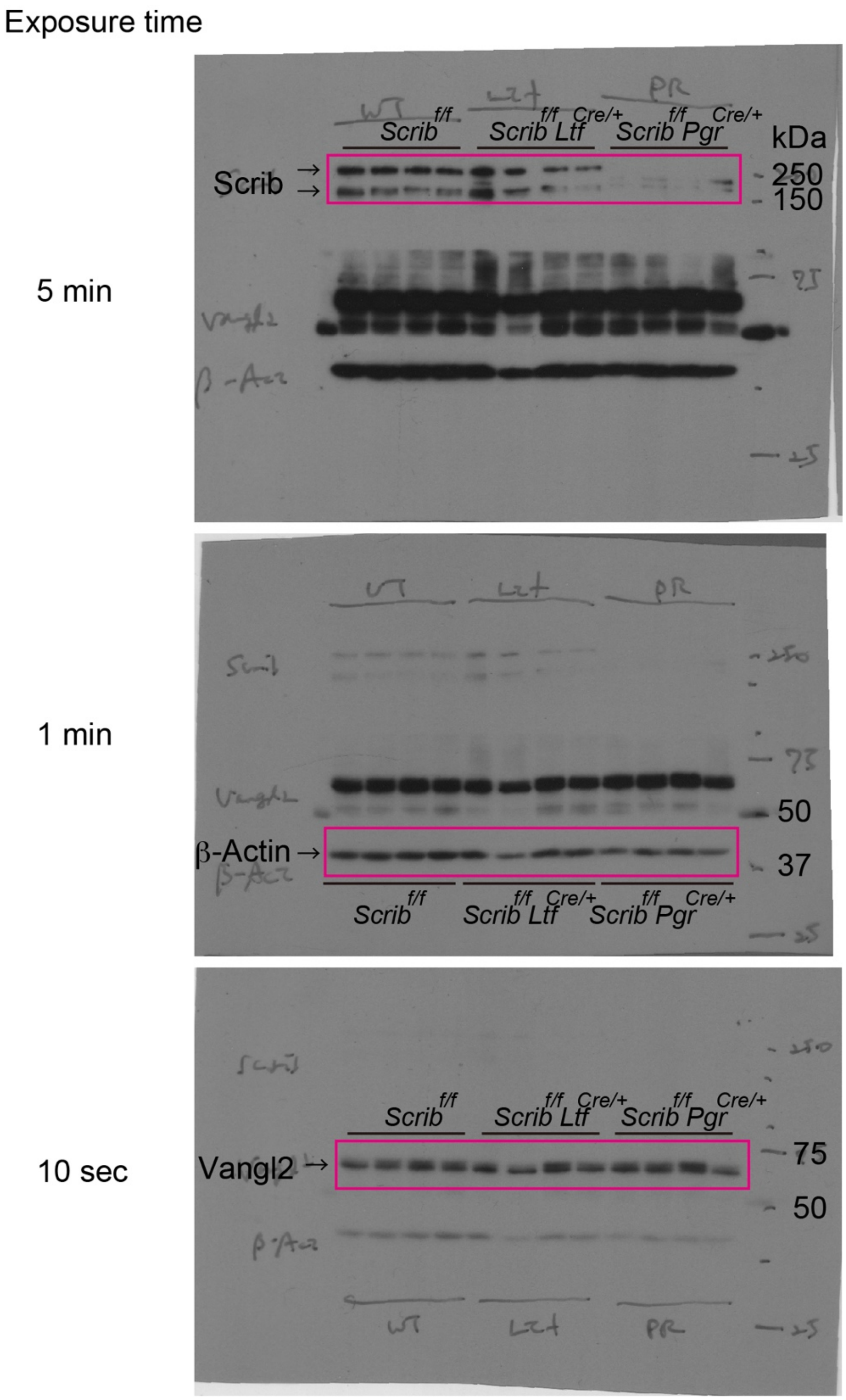

Supplementary Figure 10 l Scans of the uncropped films for Western Blotting in Supplementary Figure 1d. The same blot was processed in the different exposure times due to different sensitivities of antibodies and abundances of antigens. Bands were visualized under X-OMAT 2000 (Kodak) with Super RX-N (FUJIFILM).

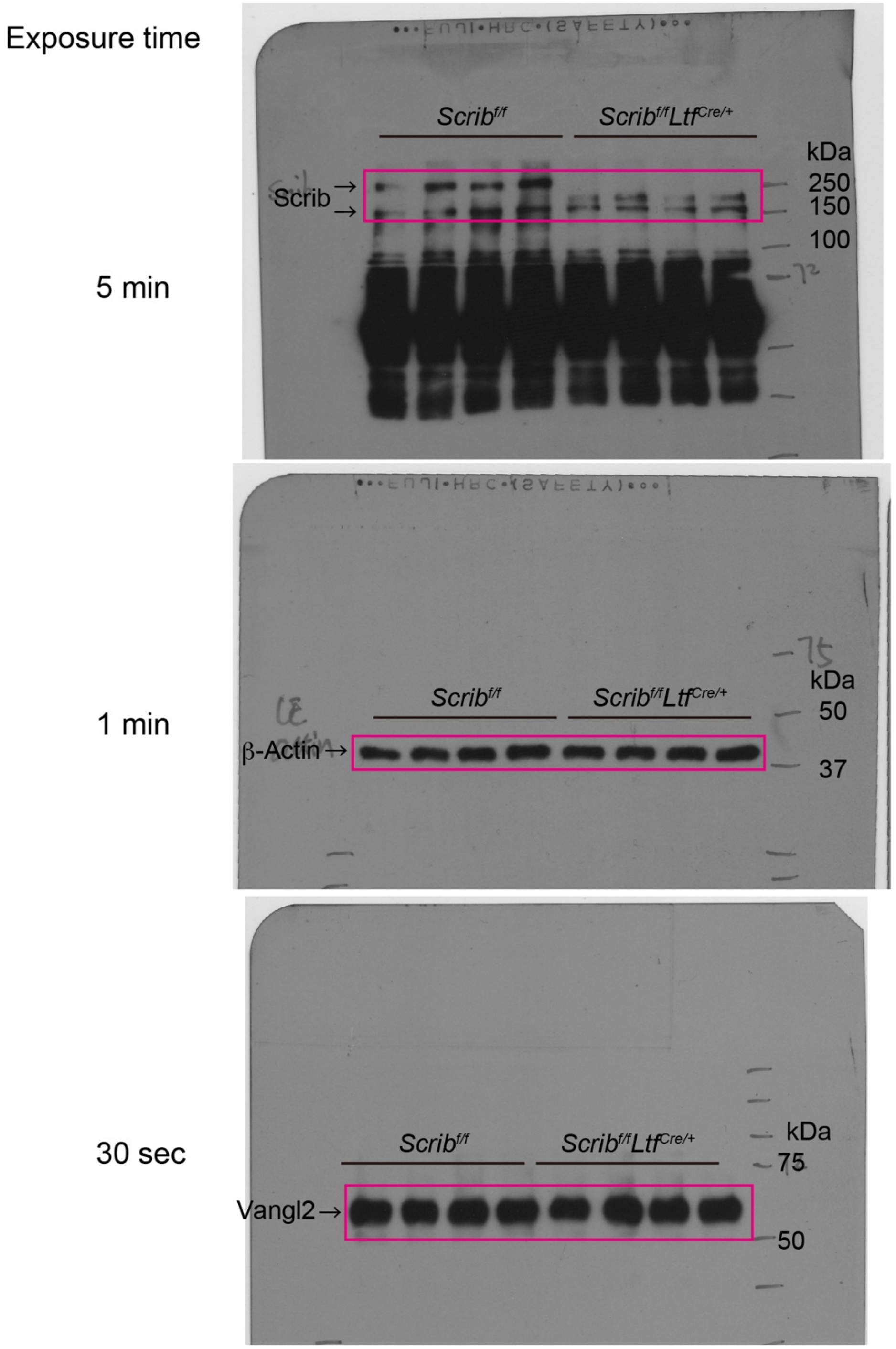

Supplementary Figure 11 | Scans of the uncropped films for Western Blotting in Supplementary Figure 1e. After development of Vangl2 bands, the membrane was reprobed for β-Actin detection. The same blot was processed in the different exposure times due to different sensitivities of antibodies and abundances of antigens. Bands were visualized under X-OMAT 2000 (Kodak) with Super RX-N (FUJIFILM).

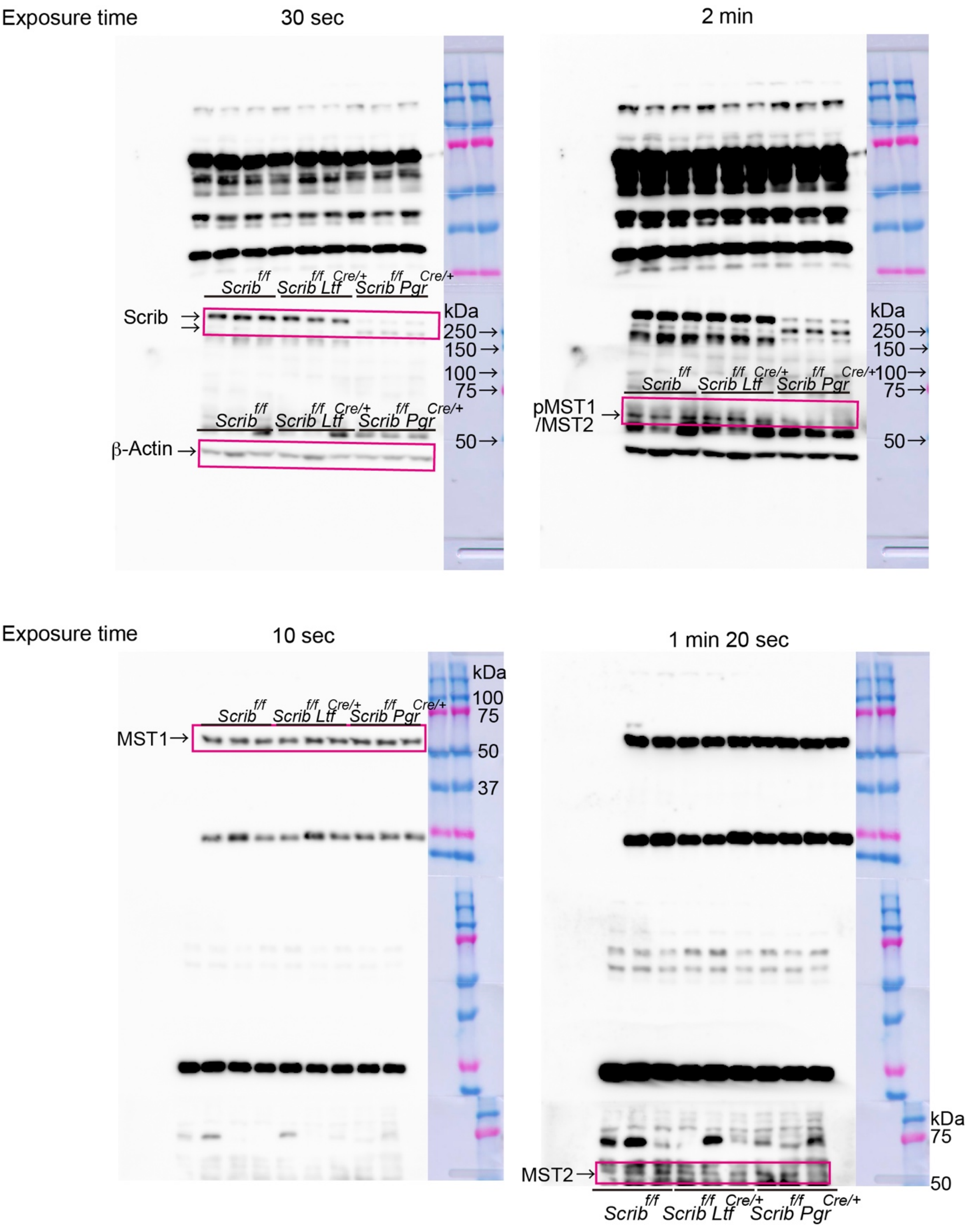

Supplementary Figure 12 | Pictures of the uncropped blots in Supplementary Figure 8c. The same samples were processed in multiple gels/blots in parallel. Bands were visualized under Amersham Imager 680 (GE Healthcare).
