# Supplementary material for: Primary decidual zone formation requires Scribble for pregnancy success in mice
Source: Nat Commun. 2019 Nov 28;10:5425. doi: 10.1038/s41467-019-13489-4 (PMC6882879; doi:10.1038/s41467-019-13489-4)
Supplement: Supplementary file 3 — Reporting Summary [file 41467_2019_13489_MOESM3_ESM.pdf]

nature research

Corresponding author(s): NCOMMS-19-20714B

Last updated by author(s): Oct 22, 2019

Reporting Summary

Nature Research wishes to improve the reproducibility of the work that we publish. This form provides structure for consistency and transparency in reporting. For further information on Nature Research policies, see Authors & Referees and the Editorial Policy Checklist.

Statistics

For all statistical analyses, confirm that the following items are present in the figure legend, table legend, main text, or Methods section.

n/a	Confirmed	
☐	☒	The exact sample size (n) for each experimental group/condition, given as a discrete number and unit of measurement
☐	☒	A statement on whether measurements were taken from distinct samples or whether the same sample was measured repeatedly
☒	☐	The statistical test(s) used AND whether they are one- or two-sided<br>*Only common tests should be described solely by name; describe more complex techniques in the Methods section.*
☒	☐	A description of all covariates tested
☒	☐	A description of any assumptions or corrections, such as tests of normality and adjustment for multiple comparisons
☐	☒	A full description of the statistical parameters including central tendency (e.g. means) or other basic estimates (e.g. regression coefficient) AND variation (e.g. standard deviation) or associated estimates of uncertainty (e.g. confidence intervals)
☐	☒	For null hypothesis testing, the test statistic (e.g. F, t, r) with confidence intervals, effect sizes, degrees of freedom and P value noted<br>*Give P values as exact values whenever suitable.*
☒	☐	For Bayesian analysis, information on the choice of priors and Markov chain Monte Carlo settings
☒	☐	For hierarchical and complex designs, identification of the appropriate level for tests and full reporting of outcomes
☒	☐	Estimates of effect sizes (e.g. Cohen's d, Pearson's r), indicating how they were calculated

Our web collection on statistics for biologists contains articles on many of the points above.

Software and code

Policy information about availability of computer code

Data collection	NIS-Elements Viewer 4.20 (ver. 4.20, Nikon)
Data analysis	Imaris (ver. 9.2.0., Bitplane), Prism6 (graphpad)

For manuscripts utilizing custom algorithms or software that are central to the research but not yet described in published literature, software must be made available to editors/reviewers. We strongly encourage code deposition in a community repository (e.g. GitHub). See the Nature Research guidelines for submitting code & software for further information.

Data

Policy information about availability of data

All manuscripts must include a data availability statement. This statement should provide the following information, where applicable:

- Accession codes, unique identifiers, or web links for publicly available datasets
- A list of figures that have associated raw data
- A description of any restrictions on data availability

The authors declare that all data supporting the findings of this study are available within the paper and its supplementary information files.

Field-specific reporting

Please select the one below that is the best fit for your research. If you are not sure, read the appropriate sections before making your selection.

☒ Life sciences ☐ Behavioural & social sciences ☐ Ecological, evolutionary & environmental sciences

For a reference copy of the document with all sections, see nature.com/documents/nr-reporting-summary-flat.pdf

Life sciences study design

All studies must disclose on these points even when the disclosure is negative.

Sample size	The sample size of each group is more than 3 since all experiments should be done at least 3 times to confirm the result. Sample size was determined to be adequate based on the consistency of measurable differences between each group.
Data exclusions	No data were excluded from the analysis
Replication	Each experiments were repeated at least 3 times and the replicate data were reliable.
Randomization	all samples were allocated at random
Blinding	Investigators were blinded to group allocation during data collection

Reporting for specific materials, systems and methods

We require information from authors about some types of materials, experimental systems and methods used in many studies. Here, indicate whether each material, system or method listed is relevant to your study. If you are not sure if a list item applies to your research, read the appropriate section before selecting a response.

Materials & experimental systems

n/a	Involved in the study	
☐	☒	Antibodies
☒	☐	Eukaryotic cell lines
☒	☐	Palaeontology
☐	☒	Animals and other organisms
☒	☐	Human research participants
☒	☐	Clinical data

Methods

n/a	Involved in the study	
☒	☐	ChIP-seq
☒	☐	Flow cytometry
☒	☐	MRI-based neuroimaging

Antibodies

Antibodies used

For immunostaining, following primary antibodies were used: Scribble (H300, SantaCruz), Vangl2 (custom made in JP Borg's laboratory, INSERM), ERα (sc-542, Santa Cruz), PR (8757, Cell Signaling Technology), E-cadherin (3195s, Cell Signaling Technology), β-catenin (sc-1496, Santa Cruz), Ki67 (RM-9106-S, Thermo Scientific), pHH3 (9701s, Cell Signaling Technology), FLK1 (RB-9239, Neomaker), CK8 (TROMA-1, Hybridoma Bank), ZO-1 (14-9776-82 thermofisher), Sav1 (#13301, Cell Signaling technology), Lats1 (#3477, Cell Signaling technology), Cleaved Caspase-3 (9661s, Cell Signaling technology) and YAP (4912s, Cell Signaling Technology). E-Cadherin (3195s, Cell Signaling Technology). To detect signals, following secondary antibodies are used: Alexa Fluor® 488 AffiniPure Donkey Anti-Rabbit IgG (H+L) (Jackson ImmunoResearch, 11-545-152), Alexa Fluor® 594 AffiniPure Donkey Anti-Rabbit IgG (H+L) (Jackson ImmunoResearch, 711-585-152), Alexa Fluor® 594 AffiniPure Donkey Anti-Rat IgG (H+L) (Jackson ImmunoResearch, 712-585-150), Cy2 AffiniPure Donkey Anti-Rat IgG (H+L) (Jackson ImmunoResearch, 712-225-153) and Cy3 AffiniPure Donkey Anti-Goat IgG (H+L) (Jackson ImmunoResearch ,705-165-147).
For Western blotting, the following primary antibodies were used: Scribble (custom made by Mireille Montcouquiol), MST1 (3682S, Cell Signaling technology), MST2 (3952S, Cell Signaling technology), pMST1/MST2 (49332S, Cell Signaling technology), CD45 (103102, biolegend/Labome) and β-Actin (sc-1615, Santa Cruz). For signal detection, the following secondary antibodies were used: Peroxidase AffiniPure Donkey Anti-Rabbit IgG (H+L) (Jackson ImmunoResearch, 711-035-152), Peroxidase AffiniPure Donkey Anti-Rat IgG (H+L) (Jackson ImmunoResearch, 712-035-150) and Peroxidase AffiniPure Donkey Anti-Goat IgG (H+L), (Jackson ImmunoResearch, 705-035-147)

Validation

Scribble (H300, SantaCruz): validated in this paper using knockout (KO) tissues. See Figure1a and Supplementary Figure 1b and c.
ERα (sc-542; Santa Cruz) : Nat Commun. 2018 Feb 9;9(1):603.
PR (8757; Cell Signaling technology): Nat Commun. 2018 Feb 9;9(1):603.
Vangl2 (custom made in JP Borg's laboratory, INSERM): PNAS, 2016; 113, E8079-E8088
pHH3 (9701s, Cell Signaling Technology): Neural Development, 2019 Mar 12;14(1):6
FLK1 (RB-9239, Neomaker): JBC, 277, 29260-29267 (2002)
ZO-1 (14-9776-82 thermofisher) : Cell Rep, 2014 Jul 24;8(2):382-92.
Sav1 (#13301, Cell Signaling technology):Oncotarget. 2019 Feb 19; 10(15): 1525–1538.
Lats1 (#3477, Cell Signaling technology): Oncotarget. 2019 Feb 19; 10(15): 1525–1538.
YAP (4912s, Cell Signaling Technology): Nat Commun. 2019 Jun 26;10(1):2797.
E-Cadherin (3195s, Cell Signaling Technology): Dev Cell. 2011 Dec 13;21(6):1014-25.
β-catenin (sc-1496, Santa Cruz): Development. 2011 May;138(10):1967-75.
CK8 (TROMA-1, Hybridoma Bank, Iowa): J Embryol Exp Morphol. 1981 Aug;64:45-60.
Ki67 (RM-9106-S, Thermo Fisher Scientific): Proc Natl Acad Sci U S A. 2016 Dec 13;113(50):E8079-E8088.
CD45 (103102, biolegend/Labome): Cell Rep. 2019 May 7;27(6):1755-1768.e4.
Cleaved Caspase-3 (9661s, Cell Signaling technology) : Cell Rep. 2015 Apr 21;11(3):358-65.

Scribble (custom made by Mireille Montcouquiol): validated in this paper using knockout (KO) tissues. See Supplementary Figure 1b and c.
MST1 (# 3682, Cell Signaling technology):Oncotarget. 2019 Feb 19; 10(15): 1525–1538.
MST2 (# 3952, Cell Signaling technology):Oncogene. 2019 Jan;38(1):120-139.
pMST1/MST2 (#49332, Cell Signaling technology):Oncotarget. 2019 Feb 19; 10(15): 1525–1538.
β-Actin (SC-1615, Santa Cruz): EMBO J. 2002 Nov 1;21(21):5766-74.

Animals and other organisms

Policy information about studies involving animals; ARRIVE guidelines recommended for reporting animal research

Laboratory animals	The background of mice were C57BL/6J and 129 mixed. Females for experiments were 8 weeks or older. All mice used in this study were housed in Cincinnati Children' hospital animal care facility with constant 12h/12h-light/dark cycle following NIH hand institutional guide lines for the animal care and use committee. Mice were provided with autoclaved laboratory rodent diet 5010 (purina) and UV light-sterilized reverse osmosis/deionized constant circulation water ad libitum. See also "Methods" in the manuscript.
Wild animals	N/A
Field-collected samples	N/A
Ethics oversight	We follow NIH hand institutional guide lines for the animal care and use committee. We used a protocol IACUC 2016-0094 approved by Cincinnati Children's Animal Care and Use Committee per NIH guidelines.

Note that full information on the approval of the study protocol must also be provided in the manuscript.
